# Bio and Nanomaterials Based on Fe_3_O_4_

**DOI:** 10.3390/molecules191221506

**Published:** 2014-12-22

**Authors:** Jia-Kun Xu, Fang-Fang Zhang, Jing-Jing Sun, Jun Sheng, Fang Wang, Mi Sun

**Affiliations:** 1 Key Laboratory of Sustainable Development of Marine Fisheries, Ministry of Agriculture, Yellow Sea Fisheries Research Institute, Chinese Academy of Fishery Sciences, Qingdao 266071, China; E-Mails: fangfang_zhang1990@163.com (F.-F.Z.); jingjingsuna@163.com (J.-J.S.); shengjun@ysfri.ac.cn (J.S.); wendywf2002@163.com (F.W.); 2 College of Food Science and Engineering, Ocean University of China, Qingdao 266003, China

**Keywords:** Fe_3_O_4_, surface modification, application

## Abstract

During the past few years, nanoparticles have been used for various applications including, but not limited to, protein immobilization, bioseparation, environmental treatment, biomedical and bioengineering usage, and food analysis. Among all types of nanoparticles, superparamagnetic iron oxide nanoparticles, especially Fe_3_O_4_, have attracted a great deal of attention due to their unique magnetic properties and the ability of being easily chemical modified for improved biocompatibility, dispersibility. This review covers recent advances in the fabrication of functional materials based on Fe_3_O_4_ nanoparticles together with their possibilities and limitations for application in different fields.

## 1. Introduction

Owing to the unique properties, such as superparamagnetism, high surface area, large surface-to-volume ratio, low toxicity, easy separation under external magnetic fields, Fe_3_O_4_ nanoparticles have enormous potential in the fields such as immobilization of biomaterials [1,2,3,4,5,6,7,8,9,10], bioseparation [11,12,13,14,15], environmental treatment [16,17,18,19,20,21,22,23], biomedical and bioengineering usage [24,25,26,27,28,29,30,31,32,33,34,35,36], and food analysis [37,38,39,40,41,42,43,44]. Various fabrication methods have been developed for the synthesis of Fe_3_O_4_ nanoparticles, including the physical methods [45,46,47], wet chemical preparation methods [48,49,50,51,52,53,54,55,56,57,58,59,60,61,62,63,64,65,66] and microbial methods [67,68,69]. Since the bare Fe_3_O_4_ nanoparticles often have poor stability and dispersity, various modification methods have been exploited to get the soluble and biocompatible Fe_3_O_4_ nanoparticles. The resulting modified Fe_3_O_4_ nanoparticles have been extensively used for various applications. In this review, the traditional and modern methods for synthesis of Fe_3_O_4_ nanoparticles are summarized; the methods for modification of Fe_3_O_4_ nanoparticles are also described. Finally, a variety of practical and potential applications as well as the corresponding limitations of the resulting Fe_3_O_4_ nanoparticles are introduced.

## 2. Methods for Preparation of Fe_3_O_4_ Nanoparticles

The outstanding potential of Fe_3_O_4_ nanoparticles has stimulated the extensive development of the synthetic technologies, which could be broadly classified into three categories: physical, chemical and biological methods. (i) Physical methods, such as electron beam lithography [45], gas-phase deposition [46], and mechanical techniques [47]. Externally controlled tools like traditional workshop or microfabrication equipment are often involved in physical methods, where are used to process materials into the desired shape and order. Although physical methods are easy to perform, it is rather difficult for them to control the particle size. (ii) Wet chemical preparation methods, such as sol-gel synthesis [48,49], oxidation method [50,51], reduction method [52], chemical coprecipitation [53,54], hydrothermal reactions [55,56], solvothermal method [57], thermal decomposition method [58], flow injection synthesis [59], electrochemical method [60,61], aerosol/vapor phase method [62], sonochemical decomposition reactions [63,64], supercritical fluid method [65,66], synthesis using nonreactors [67]. In the case of wet chemical preparation methods, relatively less energy was consumed compared with that of physical methods. Among wet chemical preparation methods, coprecipitation of Fe^3+^ and Fe^2+^ salts is a most often employed method to prepare water-borne iron oxide nanoparticles. The size and morphology of the nanoparticles can be controlled by selectively choosing the reaction media, the physical parameters of the reaction, such as precursors, reactant concentration, base (NaOH, ammonium hydroxide, and CH_3_NH_2_), ionic strength (N(CH_3_)^4+^, CH_3_NH^3+^, NH^4+^, Na^+^, Li^+^ and K^+^), reaction temperature, pH of the media, and also some other factors [68]. For instance, an increase of the mixing rate tends to decrease the particle size. Moreover, inlet of nitrogen into the reaction system that protects against critical oxidation of the magnetite also reduces the particle size when compared to methods without oxygen removal. However, coprecipitation protocol leads to reduced control of particle shape, broad distributions of sizes and aggregation of particles. In general, the size distribution of nanoparticles is an important factor to be considered for a particular application. Some of wet chemical methods can yield efficient control of the particle size by carefully adjusting the involved parameters, including sol-gel method, hydrothermal method, flow injection method, electrochemical method, sonochemical decomposition method, supercritical fluid method and synthesis using nanoreactors. (iii) Microbial method. Microbial method is an environment friendly nanoparticle formation processes which can produce 5–90 nm pure magnetite or metal-substituted magnetite without usage of toxic chemicals in their synthesis process [69,70,71,72]. Microbial method represents an advantageous manufacturing technology with respect to high yield, good reproducibility, and good scalability, as well as low costs and low energy input, but the fermentation process is rather time-consuming. Table 1 shows the summary of various methods for preparing Fe_3_O_4_ nanoparticles.

**Table 1:** Comparation between methods for synthesis of magnetic nanoparticles [46,47,73].

	Methods	Advantages	Disadvantages
Physical methods	Electron beam lithography	well controlled inter-particle spacing	expensive and highly complex machines requiring
	Gas-phase deposition	easy to perform	difficult to control the particle size
	Mechanical techniques	no chemicals involved	highly complex machines requiring and time-consuming
Wet chemical preparation methods	Sol-gel synthesis	precisely controlled in size, aspect ratio, and internal structure	weak bonding, low wear-resistance, high permeability
	Oxidation method	uniform size and narrow size distribution	small-sized ferrite colloids
	Reduction method	simple	high reaction temperature
	Chemical coprecipitation	simple and efficient	not suitable for the preparation of high pure, accurate stoichiometric phase
	Hydrothermal reactions	easy to control particle size and shapes	high reaction temperature, high pressure
	Solvothermal method	easy to control particle size and shape	high reaction temperature
	Thermal decomposition method	easy to control particle size and shape	involve multiple steps
	Flow injection synthesis	good reproducibility and high mixing homogeneity together with a precise control of the process	need continuous or segmented mixing of reagents under a laminar flow regime in a capillary reactor
	Electrochemical method	easy to control particle size	bad reproducibility
	Aerosol/vapor phase method	high yields	extremely high temperatures
	Sonochemical decomposition reactions	narrow particle size distribution	mechanism not still understood
	Supercritical fluid method	efficient control of the particle size, no organic solvents involved	critical pressure and temperature
	Synthesis using nanoreactors	precisely control the particle size	complex condition
Microbial methods	Microbial incubation	environmental friendly, high yield, good reproducibility, and good scalability, low cost	time-consuming

## 3. Modification of Fe_3_O_4_ Magnetic Nanoparticles

Because of the high surface energy, the naked Fe_3_O_4_ nanoparticles are generally unstable and aggregate easily, which strongly affects their dispersion into aqueous medium. In addition, Fe_3_O_4_ nanoparticles are highly susceptible to be oxidized to γ-Fe_2_O_3_ nanoparticles in the presence of oxygen [74]. To overcome such limitations, various surface modification methods have been developed to modify the surface of naked Fe_3_O_4_ nanoparticles via loading of other chemicals or biological materials during or after the synthesis process to improve the dispersibility, stability, biocompatibility and biodegradability for specific purposes [75,76,77,78,79,80,81,82,83,84,85,86,87,88,89,90,91,92,93,94]. With proper surface modification, the stability, dispersity and biocompatibility of Fe_3_O_4_ nanoparticles could be improved, and the oxidation process from Fe_3_O_4_ nanoparticles to γ-Fe_2_O_3_ nanoparticles could be greatly slowed down.

The common reagents employed for modification of Fe_3_O_4_ nanoparticles includes surfactants (such as oleic acid(OA) [35,75], lauric acid [76], alkane sulfonic acids [77], and alkane phosphonic acids) [78], polymers (such as polyethylene glycol (PEG) [79], polyvinylpyrrolidone (PVP) [80], poly (ethylene-co-vinyl acetate) [81], polylactic-co-glycolic acid (PLGA) [82], polyvinyl alcohol (PVA) [83], polystyrene [84], polyethyleneimine (PEI), and poly(acrylic acid) (PAA) [85]) and natural dispersants (chitosan [86,87], dextran [88], gelatin [89], polylactic acids [90], starch [91], albumin [92], liposomes [93], and ethyl cellulose [94]). The methods of modification of Fe_3_O_4_ nanoparticles mainly include physical immobilization, covalent conjugation, and biologically mediated specific interaction. The advantages and disadvantages of these three immobilization methods are summarized in Table 2.

**Table 2:** Comparation between different immobilization methods.

Methods	Interactions	Advantages	Disadvantages
Physical immobilization	physical absorption, electrostatic interaction, hydrogen bonds, van der Waals forces, and hydrophobic interactions	easy to perform and recycle, no additional coupling reagents and surface treatment are required	nonspecificity, the binding stability is highly affected by environmental conditions
Covalent conjugation	covalent interaction	the binding process can be rationally regulated with specific functional groups	nonspecificity, the support can’t be recycled
Biologically mediated specific interaction	biologically mediated specific interaction	site-specific	site-selective attachment is desired

**Scheme 1:**
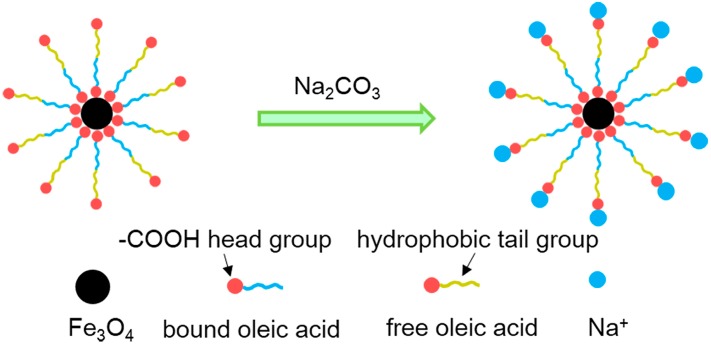
Schematic representation for interaction of oleic acid (OA) modified Fe_3_O_4_ nanoparticles with sodium carbonate. OA is chemically bound to Fe_3_O_4_ nanoparticles by the carboxyl head group (-COOH) and the hydrophobic tail group is free, making it non-dispersible in aqueous medium. The hydrophobic tail in turn interacts with the free OA via hydrophobic interactions. The formulation is stabilized in aqueous medium by ionization of the carboxyl head group of free OA by sodium carbonate, wherein Na^+^ interact by ionic interactions with COO^−^ group of free OA [75].

Jadhav *et al.* prepared oleic acid (OA) functionalized Fe_3_O_4_ nanoparticles using modified wet method, and sodium carbonate was used to improve the biological applicability (Scheme 1) [75]. In another example, Yang *et al.* synthesized PEG-coated Fe_3_O_4_ nanoparticles via traditional chemical coprecipitation method, the influence of vapor pressure, molecular weights and amounts of PEG on the structural and paramagnetic properties of PEG-Fe_3_O_4_ NPs were investigated [79]. Fe_3_O_4_ nanoparticles synthesized in sealed environment (S-Fe_3_O_4_) displayed much high crystalline quality than that synthesized in open environment (O-Fe_3_O_4_). The calculated average crystalline size of S-Fe_3_O_4_ and O-Fe_3_O_4_ is 15.2 nm and 14.5 nm, respectively. Both of the S-Fe_3_O_4_ and O-Fe_3_O_4_ nanoparticles showed superparamagnetic properties, and the saturation magnetization for S-Fe_3_O_4_ and O-Fe_3_O_4_ nanoparticles is 44 emu/g and 24 emu/g, respectively. The well-dispersed magnetic PEG-Fe_3_O_4_ nanoparticles with better size distribution can be obtained with adding 4 g PEG1000 while sealing the beaker. There were no significant size change caused by the PEG coating. However, the saturation magnetization of PEG-Fe_3_O_4_ nanoparticles showed an apparent decrease compared to that of bulk material (92 emu/g), which could be attributed to the surface disorder or spin canting at the surface of nanoparticles. Qu *et al.* prepared Fe_3_O_4_–chitosan nanoparticles with core-shell structure [87]. Oleic acid (OA) modified Fe_3_O_4_ nanoparticles (MN) were firstly prepared by coprecipitation, chitosan was then added to coat on the surface of the Fe_3_O_4_ nanoparticles by physical absorption, and glutaraldehyde was used to crosslink the amino groups on the chitosan. The saturation magnetization of the Fe_3_O_4_–chitosan nanoparticles (30.7 emu/g) was lower than the pristine Fe_3_O_4_ nanoparticles (74.3 emu/g), which could be partly attributed to the existence of the large amount of diamagnetic chitosan in the Fe_3_O_4_–chitosan nanoparticles.

## 4. Applications of Fe_3_O_4_ Nanoparticles 

Due to the unique properties, Fe_3_O_4_ nanoparticles appear to be very promising for their applications in protein immobilization, bioseparation, environments treatment, biomedical and bioengineering usage, and food analysis.

### 4.1. Protein Immobilization

Protein immobilization serves as a very effective tool to solve the difficulties encountered in the catalytic application of free enzymes, such as poor stability and hard recovery. It is of vital importance to select proper immobilization basis for protein immobilization. Fe_3_O_4_ nanoparticles have been intensively utilized to realize this objective due to its unique magnetic performance, and various practical and economical biocatalysts with improved stability and reusability have been fabricated based on Fe_3_O_4_ nanoparticles, which could be easily separated from the reaction medium in the presence of external magnetic field [1,2,3,4,5,6,7,8,9,10,95,96,97,98,99,100,101,102,103,104,105,106,107,108,109,110,111,112,113,114]. Proteins could be immobilized onto Fe_3_O_4_ nanoparticles in the manner of physical absorption [95,96,97], covalent bonding [98,99,100,101,102,103,104], and bioconjugation [105,106,107]. Coupling reagents, such as glutaraldehyde [99,100,101,102,108,109,110], 1-ethyl-3-(3-dimethylaminopropyl) carbodiimide hydrochloride (EDC) [103,104,111,112,113] and sodium tripolyphosphate (TPP) [114], are often utilized to achieve much more stable immobilization via covalent bonding because their functional groups can interact with both functional groups of the modified magnetic nanoparticles and proteins. For example, Huang *et al.* covalently bound glucose oxidase to Fe_3_O_4_/silicon dioxide nanoparticles using glutaraldehyde, resulting in an activity of immobilized glucose oxidase of 4570 U/g at pH 7 and 50 °C [102]. The immobilized glucose oxidase retained 80% of its initial activity after 6 h at 45 °C compared to only 20% for the free enzyme. After six cycles of repeated use, the immobilized glucose oxidase still maintained 60% of its initial activity; 75% of its initial activity remained after 1 month at 4 °C compared to 62% for the free enzyme. Hong *et al.* obtained amine-functionalized magnetic nanogel by Hoffman degradation of the polyacrylamide (PAM)-coated Fe_3_O_4_ nanoparticles. α-Chymotrypsin (CT) covalently bound to the magnetic nanogel with reactive amino groups by using EDC as coupling reagent [104]. The binding capacity was determined to be 61 mg enzyme/g nanogel by BCA protein assay. Specific activity of the immobilized CT was measured to be 0.93 U/(mg min), 59.3% as that of free CT. The immobilized CT still had a remaining activity of 60% when the reaction temperature rose to 60 °C while free CT lost all-initial activity. Wu *et al.* prepared magnetic Fe_3_O_4_-chitosan nanoparticles by cross-linking with TPP, precipitation with NaOH and oxidation with O_2_ in hydrochloric acid aqueous phase containing chitosan and Fe(OH)_2_ [114]. The adsorption capacity of the prepared Fe_3_O_4_-chitosan nanoparticles to lipase was 129 mg/g; and the maximal enzyme activity was 20.02 μmol·min^−1^·mg^−1^ (protein), and 55.6% activity was retained at a certain loading amount.

### 4.2. Bioseparation

Magnetic separation is a commonly used technique for polypeptide/protein separation and cell separation. Magnetic separation possesses several advantages such as timesaving, gentle, easily automated, and can be directly used to remove target compounds from crude medium by the simple application of an external magnetic field. To construct Fe_3_O_4_ based composite nanomaterials for separation, core/shell microspheres are generally fabricated with a Fe_3_O_4_ as a core and other functional materials as a shell [115,116,117,118,119,120,121,122,123]. Ma *et al.* synthesized the Fe_3_O_4_@mTiO_2_ microspheres with a well-defined core/shell structure, the high specific surface area (167.1 m^2^/g), large pore volume (0.45 cm^3^/g), appropriate and tunable pore size (8.6–16.4 nm), and high magnetic susceptibility [123]. The composite could selectively enrich phosphopeptides from complex mixtures even at a very low molar ratio of phosphopeptides/non-phosphopeptides (1:1000), large enrichment capacity (as high as 225 mg/g, over 10 times as that of the Fe_3_O_4_@TiO_2_ microspheres), extreme sensitivity (the detection limit was at the fmol level), excellent speed (the enrichment can be completed in less than 5 min), and high recovery of phosphopeptides (as high as 93%).

**Scheme 2:**
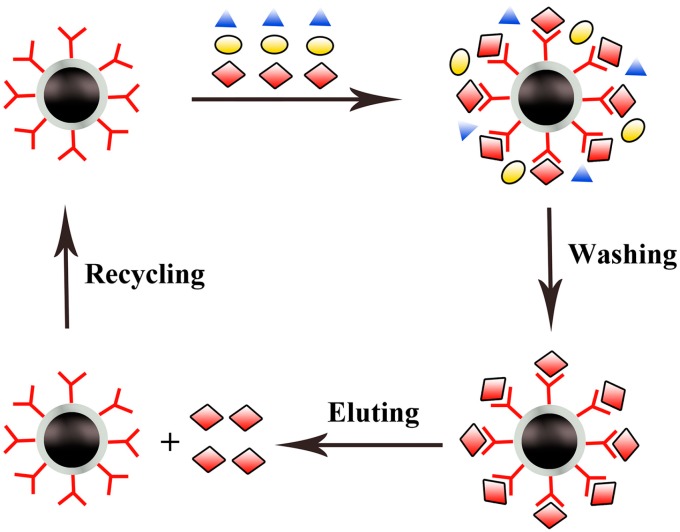
Illustration of the separation mechanism of affinity chromatography utilizing Fe_3_O_4_ based composite as packing material.

To realize more effective separation of protein, affinity chromatography utilizing Fe_3_O_4_ based composites as packing materials is usually taken into account [124]. Several functionalized Fe_3_O_4_ nanoparticles have been strategically developed for the purification of specific proteins utilizing the affinity interactions. The target protein is firstly captured by modified Fe_3_O_4_ nanoparticles from crude samples to form a complex, and the captured target protein on the particles is subsequently eluted from metal ion chelated magnetic nanoparticles by applying buffer solution with different pH or ion strength (Scheme 2). These metal ion chelated magnetic nanoparticles were usually regenerated by using EDTA to strip the adsorbed protein and metal ion and then reloading with metal ion for recycling.

### 4.3. Environmental Treatment

**Scheme 3:**
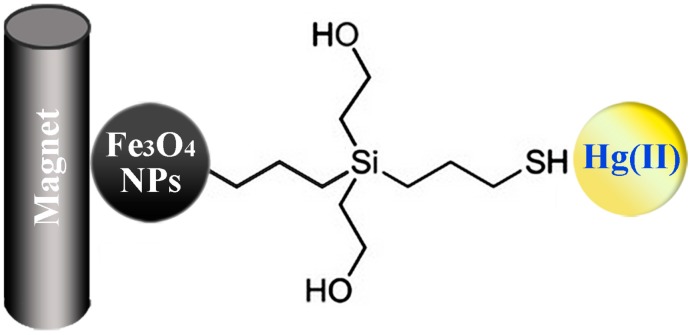
Illustration of the moval and recovery of Hg(II) using thiol-functionalized mesoporous silica-coated magnetite nanoparticles [129].

As a result of rapid industrialization and urbanization, various pollutants particularly those entering aquatic systems have attracted worldwide concern. The development of efficient and cost-effective methods for environmental treatment is of primary concern for sustainable economic and social development. Due to the extremely small particle size, high surface-area-to-volume ratio, and more important the magnetism, Fe_3_O_4_ nanoparticles have been widely used and have shown promising performance in environments treatment, including pollutant removal and toxicity mitigation [16,17,18,19,20,21,22,23,125,126,127,128,129]. Proper surface coating cannot only improve the removal capacity and affinity of the Fe_3_O_4_ nanoparticles, but also promote the desorption process. Pollutants generally adsorb to the surface of Fe_3_O_4_ nanoparticles through interactions including physical adsorption, ion-exchange, chemical bonding (complexation and/or chelation), hydrogen bonds, and van der Wall forces. Fe_3_O_4_/ZrO_2_/chitosan composite was synthesized and employed for the removal of amaranth and tartrazine dyes removal, the adsorption capacities of which were 99.6 and 47.3 mg/g for amaranth and tartrazine dyes, respectively [128]. In another report, Hakami *et al.* prepared Fe_3_O_4_ nanoparticles functionalized with thiol groups by adding (3-mercaptopropyl) trimethoxysilane on silica-coating to remove Hg, and the sulfur atoms in thiol moieties served as ligands to bind with soft metal cation Hg^+^. Thiourea was added to facilitate desorption of Hg because of the presence of sulfur atoms (Scheme 3) [129].

In the real engineering applications, the strategically utilization of Fe_3_O_4_ nanoparticles should consider the complex environmental conditions such as background ions, humic substances, temperature, and pH. 

### 4.4. Biomedical Usage

Fe_3_O_4_ nanoparticles with appropriate surface properties have been widely used for numerous biomedical and bioengineering applications such as targeted drug delivery, biosensor, magnetic resonance imaging, hyperthermia, tissue engineering, magnetofection, *etc.* All these applications require that these nanoparticles not only possess high magnetization values, but also with narrow particle size distribution and similar surface topography, so that the particles have uniform physical and chemical properties. Moreover, the magnetic nanoparticles for biomedical applications should be non-toxic and biocompatible. In a word, both of the nature and the geometric arrangement of surface coatings on the nanoparticles have apparent influence on bio-kinetics and bio-distribution of nanoparticles in the body.

#### 4.4.1. Targeted Drug Delivery

Due to the unique capabilities (e.g., superparamagnetism and biocompatibility) and the negligible side effects, magnetic Fe_3_O_4_ nanoparticles with proper surface modification and conjugated targeting ligands/molecules have become a major research focus for drug delivery applications. Compare to the conventional, non-targeted methods of drug delivery, magnetic nanoparticles are promising drug carriers due to the better specificity to the target site and the reduced adverse effects. Drug carried by magnetic nanoparticles could be concentrated at the desired site to receive much high therapeutic efficiency. Since the drugs simply physically attached to the nanoparticle surface tend to release quickly before reaching the final destination, a core-shell structure consisting of a magnetic core and a shell is preferred in magnetic drug-delivery systems to achieve sufficient drug loading capacity and good transportation effect [130,131,132,133,134,135,136,137,138]. For example, Chen *et al.* prepared Fe_3_O_4_*@*SiO_2_ core–shell nanoparticles and grafted a widely used anticancer agent doxorubincin (DOX) to the surface of the core–shell nanoparticles via an amide bond with the aid of a spacer arm. Most of the conjugated DOX can release from the nanoparticles within 12 h and the release process prefers low pH conditions. The saturation magnetization value of the obtained superparamagnetic DOX-grafted Fe_3_O_4_*@*SiO_2_ core-shell structure nanoparticles was 49.3 emu·g^−1^, indicating its great potential application in the treatment of cancer using magnetic targeting drug-delivery technology [136].

#### 4.4.2. Biosensor

**Scheme 4:**
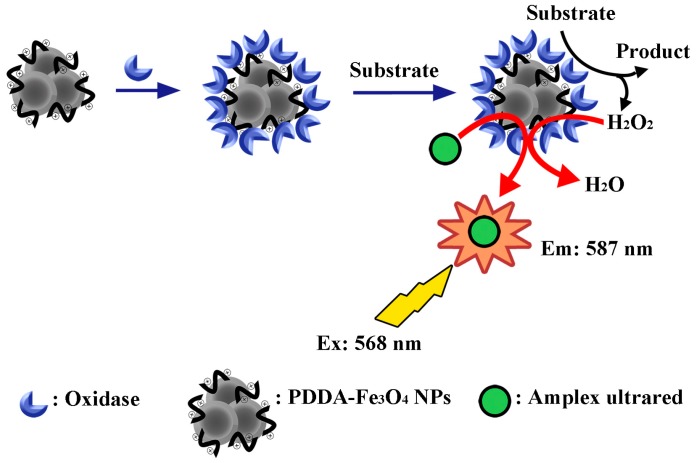
Illustration of Fe_3_O_4_ nanoparticles play a peroxidase-like role to detect the specific substrate in the presence of AU [148].

Fe_3_O_4_ nanoparticles based bioanalytical sensors could be fabricated by coating Fe_3_O_4_ nanoparticles with materials such as a fluorescent one [139,140], a metal [141,142], silica [143,144], or a polymer [145,146]. Tang *et al.* developed a practical glucose biosensor by combining the intrinsic peroxidase-like activity of Fe_3_O_4_ nanoparticles and the anti-interference ability of the nafion film. Glucose oxidase was simply mixed with Fe_3_O_4_ nanoparticles and cross-linked on the Pt electrode with chitosan medium by glutaraldehyde, and then covered with a thin nafion film. The biosensor showed high sensitivity (11.54 μAcm^−2^·mM^−1^), low detection limit (6 × 10^−6^ M), and good storage stability [147]. Liu *et al.* developed a reusable, single-step system for the detection of specific substrates using oxidase-functionalized Fe_3_O_4_ nanoparticles as a bienzyme system and using amplex ultrared (AU) as a fluorogenic substrate. A composite of poly (diallyldimethylammonium chloride)-modified Fe_3_O_4_ nanoparticles and oxidase was prepared for the quantification of specific substrates through the H_2_O_2_-mediated oxidation of AU. The reaction process was monitored by checking fluorescence intensity at 587 nm, and the minimum detectable concentrations of glucose, galactose, and choline were found to be 3, 2, and 20 μM utilizing glucose oxidase-Fe_3_O_4_, galactose oxidase-Fe_3_O_4_, and choline oxidase-Fe_3_O_4_ composites, respectively (Scheme 4) [148].

#### 4.4.3. Magnetic Resonance Imaging

Magnetic resonance imaging (MRI) is a commonly used non-invasive medical imaging technique in clinical medicine to visualize the structure and function of tissues, which is based on the behavior, alignment and interaction of protons in the presence of an applied magnetic field [149,150,151,152,153]. For example, Fan *et al.* present a relatively simple and scalable approach for preparing poly(poly(ethyleneglycol) monomethacrylate) (P(PEGMA))-grafted Fe_3_O_4_ core–shell nanoparticles with well-controlled properties using a solvent-free ATRP [153]. The so-obtained P(PEGMA)-grafted nanoparticles have a uniform hydrodynamic particle size of 36.0 ± 1.2 nm. The morphology and viability of the macrophage cells cultured in a medium containing 0.2 mg/mL of P(PEGMA)-grafted nanoparticles were found similar to those of cells cultured without nanoparticles, indicating an absence of significant cytotoxicity effects. T2-weighted magnetic resonance imaging (MRI) of P(PEGMA)-grafted MNPs showed that the magnetic resonance signal is enhanced significantly with increasing nanoparticle concentration in water. The *R_1_* and *R_2_* values (longitudinal and transverse relaxivities) per millimole Fe, and *R_2_*/*R_1_* value of the P(PEGMA)-grafted MNPs were calculated to be 8.8 mM^−1^·s^−1^, 140 mM^−1^·s^−1^, and 16, respectively. These results indicate that the P(PEGMA)-grafted nanoparticles have great potential for application in MRI of specific biotargets.

#### 4.4.4. Hyperthermia

Magnetic induction hyperthermia means the exposition of cancer tissues to an alternating magnetic field, in which heat is generated due to magnetic hysteresis loss. Cancer cells exposed to magnetic particles will heat up to a temperature higher than 43 °C, at which the cancer cells are destroyed whereas the normal cells can survive. Much research work using magnetic particles for hyperthermia have manifested a therapeutic effect on several types of tumors [30,31,32,33,154,155,156]. For example, Ghosh *et al.* synthesized Fe_3_O_4_ magnetic nanoparticles (MN) capped with either oleic acid (Fe_3_O_4_-OA-MN) or polyethylene glycol (Fe_3_O_4_-PEG-MN), which were prepared by a co-precipitation method. The average particle sizes of the obtained Fe_3_O_4_-MN, Fe_3_O_4_-OA-MN and Fe_3_O_4_-PEG-MN were found to be 12, 6 and 8 nm, respectively. A 35% increase of killing effect was observed in human breast cancer cells (MCF7) after Fe_3_O_4_-OA-MN treatment, which was further enhanced (65%) in the presence of induction heating. However, only 5%–10% killing was achieved while Fe_3_O_4_-MN or Fe_3_O_4_-PEG-MN was used to treat MCF7 cells after induction heating. The effect of only OA (0.088 mg·mL^−1^, a concentration low than that in Fe_3_O_4_-OA-MN) or PEG (0.1 mg·mL^−1^) with/without induction heating on cell viability experiments indicated that loss of viability by OA was ~75%, which was higher than 1 mg of Fe_3_O_4_-OA-MN (35%) alone. However, PEG at this concentration did not show any significant change in cell toxicity. The same control experiments conducted under induction heating showed insignificant change in cell viability. These results displayed the surface characteristics of the modified magnetic nanoparticles (e.g., lipophilicity) greatly influence their hyperthermia applications in cancer therapy [156].

#### 4.4.5. Tissue Engineering

Tissue engineering is a promising technology for overcoming the organ transplantation crisis, and the fabricated tissue equivalents may also be used to screen the effects of drugs and toxins [157,158]. It has been a great challenge for scientists and medical experts to fabricate functional organs of the similar architectures *in vitro* to the *in vivo* organs, in which the cells are allocated precisely. To realize this objective, three-dimensional constructs (scaffolds or hydrogels) functioning similarly as under *in vivo* conditions should be firstly built up [159,160,161]. The cells generally isolated from a tissue biopsy, cultured *in vitro*, subsequently seeded into the three dimensional constructs. To achieve an efficient cell seeding and to enable controlled tissue assembly and complex tissue formation, magnetic force-based tissue engineering technique is required to provide magneto-responsive features to the cells [162,163,164]. The inclusion of magnetic particles has no significant effect on the porosity, stability and wetting properties of the composite scaffolds, making them appropriate for cellular support and cultivation. For instance, Sapir *et al.* created a stimulating microenvironment by inserting magnetically responsive Fe_3_O_4_ nanoparticles into a macroporous alginate scaffold, which was suitable for promoting endothelial cell organization into capillary-like structures *in vitro* [165].

#### 4.4.6. Magnetofection

Magnetofection rely on the delivery of nucleic acids (e.g., DNA, antisense oligodeoxynucleotides (AODN), and small interfering ribonucleic acids (siRNA) into the targeted cells in presence of a magnetic field [46]. The delivery of nucleic acids using viral vectors is called transduction, whereas the delivery using nonviral vectors is termed transfection. The negatively charged nucleic acids generally interact with MNPs chemically modified by cationic substances such as PEI or protamine sulfate polymers [166], which could contribute to the intracellular penetration. The application of an external Fe_3_O_4_ magnetic field directs viral or non-viral gene delivery vectors facilitates fast and efficient nucleic acid delivery towards the target cells [167].

Although substantial progress has been made with creating proper delivery systems for nucleic acids, our knowledge of the internal operation mechanism inside cells is still unclear, and target delivery of nucleic acids still has not lived up to its potential clinical application. The processes governing nucleic acid uptake and delivery are far from being clarified, as well as their intracellular interactions, intracellular trafficking and the regulation of nucleic acid action inside cells [46]. 

### 4.5. Food Analysis

**Scheme 5:**
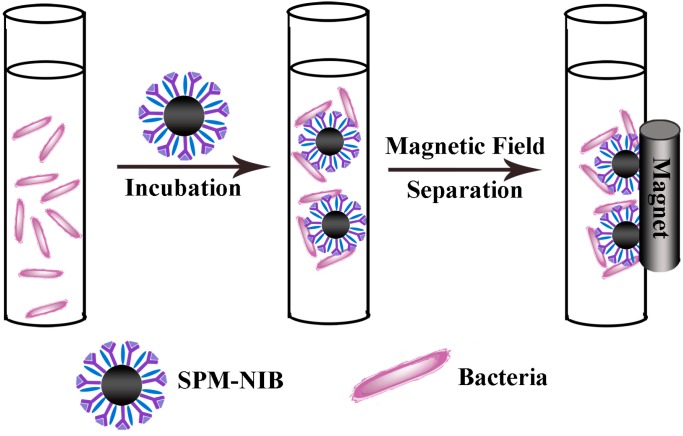
Illustration of the process of target bacteria separation using superparamagnetic nano-immunobeads (SPM-NIBs) [41].

It is of vital importance to accurately analyze food components and food contaminants for ensuring food safety and quality. Although the frequently employed techniques (e.g., gas chromatography (GC), culture and colony counting, immunoassay, high-performance liquid chromatography (HPLC), and liquid chromatography coupled with tandem mass spectrometry (LC-MS/MS)) are of very great usage for food analysis, most of them are laborious, complex, time-consuming, expensive, and show somewhat dissatisfying specificity and detectability to some special targets. Magnetic nanoparticles such as Fe_3_O_4_ are of special interest for food analysis not only because the unique properties such as low toxicity, good biocompatibility, large specific surface area, high capacity for charge transfer and convenient separation from a reaction mixture with an external magnetic field, but also for the rapid, highly selective and sensitive detection of food contaminants and food components after the proper surface modification. Fe_3_O_4_ nanoparticles are usually involved in detection techniques for food analysis in two ways: electrode modifier and sample pre-concentrator [37,38,39,40,41,42,43,168]. Fe_3_O_4_ nanoparticles have been widely used in many detection techniques for food analysis, including PCR, immunoassay, HPLC, LC-MS/MS, and optical method. For example, Liu *et al.* developed a superparamagnetic nano-immunobeads (SPM-NIBs) by conjugation of Fe_3_O_4_ nanoparticles with specific antibodies (Scheme 5). The prepared SPM-NIBs showed superior colloidal stability and reversible magnetic response to *Vibrio parahaemolyticus*, a main foodborne pathogenes from contaminated seafood. About 80% of *Vibrio parahaemolyticus* cells could be captured when the concentration of the broth was 10^3^ CFU/mL [41].

## 5. Concluding Remarks and Prospects

Due to the unique properties (e.g., superparamagnetism, high surface area, large surface-to-volume ratio, low toxicity, and easy separation), Fe_3_O_4_ nanoparticles have emerged as ideal frame materials for generating functional materials of different surface architecture, which have already displayed promising effects in practical applications in protein immobilization, bioseparation, biomedical science, environmental treatment, and food analysis. Various Fe_3_O_4_ based nanoparticles have already realized their practical applications. However, there are still many Fe_3_O_4_ based nanoparticles having not been scaled up from the laboratory scale into industry-level, and several crucial scientific, technical, and economical issues still need to be settled. Therefore, more and more efforts are still required to meet the tremendous demands for advanced materials of modern technology, which might call for interdisciplinary cooperation of material, chemistry, physics, medicine, and other related disciplines. 
